# A step forward in genetic counselling: defining practice and ethics through the Genetic Counselling Practice Consortium in Hong Kong

**DOI:** 10.1038/s10038-025-01321-5

**Published:** 2025-03-14

**Authors:** Desiree M. S. Tse, Brian H. Y. Chung, Hong Kong Genetic Counselling Practice Consortium, Hong Kong Genome Project, Annie T. W. Chu

**Affiliations:** 1 Hong Kong Genome Institute, Hong Kong, China; 2 Department of Paediatrics and Adolescent Medicine, Li Ka Shing Faculty of Medicine, The University of Hong Kong, Hong Kong, China

**Keywords:** Social sciences, Health care

## Abstract

Genetic counselling plays a crucial role in the genomic era, assisting in disease risk determination, diagnosis and management. The lack of an accredited local training program for genetic counselling in Hong Kong has led to pragmatic on-the-job training and diverse practice models. In view of the needs for enhanced awareness in genomic counselling practices among healthcare professionals, a collaborative effort - the Hong Kong Genetic Counselling Practice Consortium - was initiated to develop genomic medicine in Hong Kong. A thematic analysis of genetic counselling practice across 15 regions was conducted, revealing a broad consistency in the scope of duties, with minor differences due to social and cultural influences. Genetic counsellors generally follow a similar protocol, but some approaches vary. Ethical considerations for genetic counsellors are discussed, highlighting their responsibility towards themselves, colleagues, clients, and society. The scope of practice and code of ethics were developed to highlight the key areas of practice duties; guide the conduct of genetic counsellors; and support local counsellors in their professional training, ultimately contributing to the advancement of genomic science and health benefit of the people of Hong Kong.

## Global scene of genetic counselling as a profession

Genetic counselling (GC), a term coined by Sheldon Reed in 1947 (ref. [1]), is defined as “the process of helping people understand and adapt to the medical, psychological and familial implications of genetic contributions to disease” by the National Society of Genetic Counselors (NSGC) of the United States (US) [2]. While GC has existed in various forms for longer, it began to gain recognition as a regulated profession in the 1990s. This shift transformed GC from a service primarily provided by medical physicians into a distinct allied health profession through professionalisation, expanded scope, integration of advanced genomic technologies, emphasis on patient-centred care, interdisciplinary collaboration, and increased public awareness. This important development verifies the unique role genetic counsellors have as translators and communication bridges to convert complex genetic and genomic information to layman-friendly and relevant information to patients and their families, whilst providing psychosocial support and tailored care for them.

As the medical and scientific fields transition into the genomic era, GC has extended its application from genetic conditions to include multifactorial diseases and common conditions, encompassing both high-risk populations, and seemingly healthy individuals. Advances in genome-wide association studies (GWAS) and techniques like circulating free DNA (cfDNA) enable the assessment of disease risk, diagnosis and prognosis, and the choice and prioritisation of treatment options. With the integration of preemptive pharmacogenetic data into clinical use, the time for precision medicine with a preemptive genotyping approach has arrived. Guidelines have been established to prevent adverse drug reactions and ensure effective treatments based on the association between variants in the gene coding for drug responses [3]. Actionable genetic variants identified through genetic testing could guide the prescription of common medications, such as the lipid-lowering drug Simvastatin, enhancing patient care [4]. Despite these advancements, the understanding and dissemination of genetic information remain limited in clinical and other healthcare settings. This underscores the essential role of genetic counsellors as a communication bridge/channel among clinicians, laboratory scientists, genome curators, bioinformaticians, allied-health professionals, patients and their carers.

## Current landscape and gaps of service, prompting the establishment of the Genetic Counselling Practice Consortium in Hong Kong

In Hong Kong, cultural attitudes towards genetics and the structure of the healthcare system present significant challenges for GC services. Traditional values often prioritise family reputation, leading to a reluctance to openly discuss genetic issues, which contrasts with more individualistic cultures, where GC is more readily accepted [5, 6]. Additionally, language barriers due to the bilingual nature of the region (Cantonese and English) complicate effective communication, making it challenging for counsellors to ensure that patients fully understand complex genetic information [5, 7]. The public healthcare system, while robust, might not prioritise GC as a standard service, resulting in disparities in access and quality based on patients’ socio-economic status [6]. Compounding these issues is a general lack of public knowledge about GC practices, which deters individuals from seeking services. Limited educational outreach aimed at healthcare professionals further exacerbates the problem, leading to fewer referrals and underutilisation of GC services. Moreover, the rise of direct-to-consumer genetic testing (DTCGT) introduces challenges such as misinformation and the need for genetic counsellors to assist in interpreting results, a situation that differs from regions with more regulated DTCGT frameworks [8]. Rapid advancements in genomic technologies add another layer of complexity, highlighting the urgent need for professional development in this evolving field.

Hong Kong’s development in GC practice lags behind that of western countries/regions. In the past decades, the service delivery and coordination were fragmented. Comprehensive genetic testing and counselling services have been mainly provided by the Department of Health (DH), the Hospital Authority (HA), universities, research programmes, charitable funds and private sectors. The absence of standardised protocols across different services results in variability in the quality and comprehensiveness of care. Patients may experience different levels of service based on the clinic they visit. From 2014 to 2018, the Clinical Genetic Service (CGS) of the DH helped an annual average of approximately 5,100 patients/families with genetic conditions through three GC clinics [9]. While collaborations with universities have facilitated some advancements, ensuring that research translates into clinical practice remains a challenge, particularly in aligning educational curricula with clinical needs. Genetic services were also provided by the HA on an independent basis, driven by individual clinical practitioners, local needs and/or laboratory initiatives, resulting in a wide variation in service types and limited standard protocols. Recognising the needs for enhanced multi-disciplinary care for patients and families with a genetic conditions, DH and HA have recently transitioned the CGS’s services, including GC, to the HA’s Clinical Genetic Service Unit (CGSU) from Mid-2023 (ref. [10]). Additionally, other genetic services in Hong Kong include charitable and optional GC provided by family cancer support groups and DTCGTs, respectively.

As of today, there is no accredited and board-certified local training programme to link with a registration system for GC in Hong Kong. This lack of a structured educational pathway has led to services being provided primarily by on-the-job trained medical personnel, including clinicians, nurses, laboratory staff and experienced researchers, who are recruited to meet the pragmatic service needs of specific clinics/medical departments, e.g., obstetrics and gynaecology, familial cancer and prenatal clinics. Consequently, healthcare professionals practising GC in Hong Kong have varied GC training backgrounds and adopt diverse practice models, resulting in differences in the length of training, qualifications of trainers, curriculum specifics, clinical training opportunities and ongoing professional development, making it challenging to ensure a uniform standard of care.

As the medical and scientific fields swiftly evolve into the genomic era, a surge of service needs is foreseeable. It necessitates continuous professional development and adaptation of practices in GC. As genomic testing becomes more prevalent, the demand for skilled genetic counsellors is expected to rise significantly. In response, the Hong Kong Government has supported the launch of the Hong Kong Genome Project (HKGP) through the Hong Kong Genome Institute (HKGI) to act as a catalyst and facilitate the engagement and training of genetic counsellors [11]. Fully funded by the HKGI, the HKGP has already recruited and supported the hiring and training of at least 20 part-time and full-time genetic counsellors [11]. By aligning practice training and fostering continuous engagement these genetic counsellors working in different Partnering Centres, the HKGP has successfully implemented high standards for informed consent and comprehensive pre-test and post-test GC protocols.

**Fig. 1:**
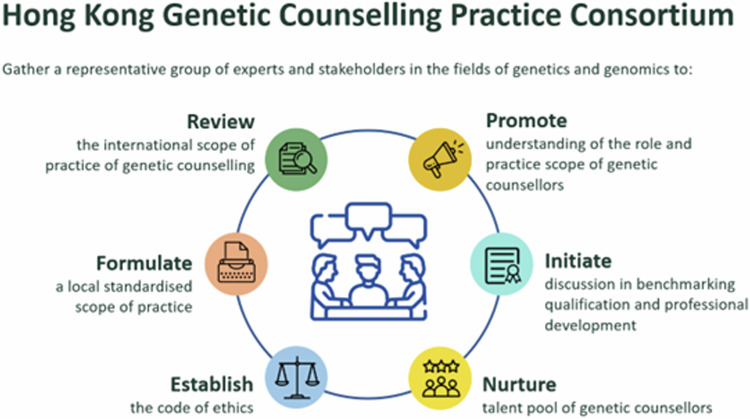
Overview of the Hong Kong Genetic Counselling Practice Consortium’s primary objectives in strengthening genetic counselling practices and enhancing patient care in Hong Kong

**Fig. 2:**
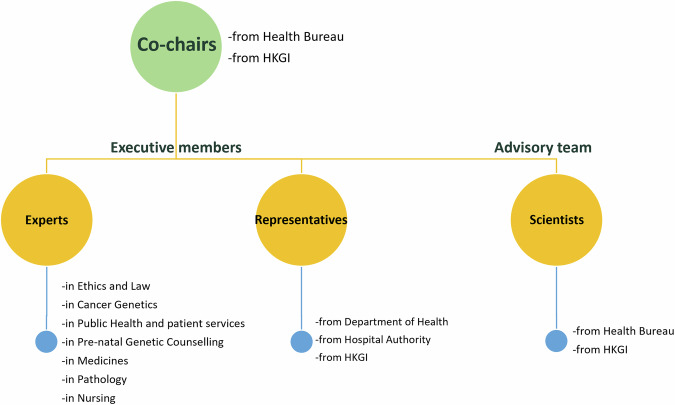
The hierarchical organisational structure of Hong Kong Genetic Counselling Practice Consortium responsible for genetic counselling initiatives

The lack of a legitimate and professionally accountable body to govern GC practice contributes to the inconsistencies in service provision and hinders the establishment of ethical standards and accreditation systems. As genomic science advances and becomes the standard of care, there is a pressing need for Hong Kong to establish a legitimate and professionally accountable body, to lead the structured development of GC practices. The entity would promote the understanding of the role and practice scope of genetic counsellors among healthcare professionals and the public. It would address the need for a structured framework to guide practice, including a code of ethics and accreditation, which is critical for enhancing the credibility and effectiveness of GC services in Hong Kong. The HKGI sees the lack of formally trained GC talents as major hindrance, and, therefore, prioritises the need to nurture them in genomic medicine. To address these challenges, a consortium pooling resources from a group of renowned medical experts working in the genetic and genomic fields is pivotal to fostering the professional development of GC services in Hong Kong (Fig. 1). The HKGI is mandated to implement the HKGP and drive the collaboration of existing infrastructure and expertise, guided by the Health Bureau (HHB; formerly known as the Food and Health Bureau). The formation of the Hong Kong Genetic Counselling Practice Consortium (Consortium) is a step towards pooling expertise and resources to meet the needs, challenges and aspirations for the development of GC practice in Hong Kong. The Consortium gathers a representative group of experts and stakeholders in the industry of genetics and genomics such as cancer genetics, public health, prenatal genetics, medicines, pathology and nursing to steer the development of GC in the local healthcare setting (Fig. 2 and Supplementary Table S1). With the mission of improving patient care through high-quality GC services, the Consortium aims to enhance the development of GC in the region by formulating the practice scope; establishing the code of ethics; designing a model of practice; and facilitating the development and implementation of a GC accreditation system.

## Formulating the scope of practice and code of ethics for genetic counselling in Hong Kong using thematic analysis

Based on the relevant publications and publicly available websites, including existing guidelines, ethical frameworks and regulatory documents pertaining to genetic counselling, we conducted a qualitative research to analyse the collected literature [12]. Led by the research question on global trends in GC practice scope and ethical conducts, a thematic analysis with an inductive approach was conducted to focus on synthesising key themes that emerge from literature sources. Key documents, including national and international guidelines on genetic counselling, ethical codes from professional organisations and recent scholarly articles, were reviewed extensively. Information was captured for initial impressions and insights related to the scope of practice and ethical considerations in genetic counselling. The literature was scrutinised for subjects or topics that would serve as a hegemonic idea or an organising principle for a theme (see Table 1 for details of the analytic steps). The data were coded systematically and independently by two research assistants. Each document was reviewed line-by-line to identify significant phrases, concepts and ideas relevant to the research objectives. To ensure inter-rater dependability, the codes were compared and revised. Significant concepts and ideas were coded to identify preliminary themes. The codes were then examined for patterns and relationships to identify broader themes. Similar codes were grouped to form preliminary themes that encapsulated shared concepts. For example, codes related to obtaining family medical history and constructing pedigree were aggregated under a broader theme of “assessing risk”. To ensure validity, themes were refined and checked against the collected documents. The final codebook identified six themes in the analysis for the scope of practice (SOP): pre-assessment, assessing risk, ordering laboratory/genomic tests, discussing clinical implications, making referrals to support services and other duties; four were found for code of ethics (COE): to self, clients/patients, colleagues and society. A final review of all themes and their definitions was conducted to ensure coherence and completeness. The themes were synthesised into a cohesive framework that outlines the scope of practice and ethical guidelines for the GC profession. Table 2 presents the 9 main themes and 22 subthemes.

**Table 1:** Description of analytic steps

Analytic steps suggested by Braun and Clarke [19]	Analytic steps of the current study
Familiarising yourself with your data	The authors have read and re-read relevant materials and some are active practitioners in the field.
Generating initial codes	Initial considerations and reflections were used to identify patterns pertaining to themes. The literature that was studied, rather than the studies’ objectives, was the data-driven focus.
Searching for themes	Themes and sub themes were used to group the codes. The relevance of the themes and sub-themes for the objectives of the investigations was examined.
Reviewing themes	The first topics that shared a particular pattern of similarities were combined into one theme after being critically analysed once again to ascertain their fundamental significance.
Defining and naming themes	Using appropriate titles to describe the central idea of the theme.
Producing the report	The authors converse on the themes and how to best describe them in a manuscript.

**Table 2:** Themes and subthemes

	Theme	Subtheme
Scope of practice	Pre-assessment	Building rapport Obtaining informed consent
	Assessing risk	Collecting medical history Discussing genetic/medical conditions/diseases
	Ordering laboratory/genomic tests	Facilitating tests Interpreting test results
	Discussing clinical implications	Explaining clinical significance Evaluating risk of recurrence Promoting psychological adaptations
	Making referrals to support services	Referring patients to physical health services Referring patients to psychological support
	Other duties	Involving in research Developing public health policies Advocating public education
Code of ethics	To self	Duty of candour Professional indemnity arrangements
	To clients/patients	Duty of care Communication, consent and confidentiality Record keeping Minimising risk
	To colleagues	Maintaining boundaries Minimising risk
	To society	

## Review of the scope of practice and code of ethics in regions providing genetic counselling services

A SOP outlines the areas in which a practitioner possesses the particular knowledge, skills and experience to practise lawfully, safely, and effectively, while meeting professional standards and ensuring public safety [13]. A well-defined SOP is crucial for maintaining high-quality GC services by applying evidence-based professional practice [14]. Complementing the SOP, a code of ethics (COE) provides guidelines that clarify and uphold the profession’s goals, values and standards. The COE helps practitioners act ethically and be accountable in their professional duties. A global review of SOPs and COEs can inform the development of these frameworks in local contexts, allowing for the integration with our unique societal and cultural factors of the community. While COEs for genetic counsellors worldwide share many similarities, they also reflect differences in their emphasises due to cultural or regulatory variations. While recognised professions are bound by the laws with their SOPs set by licensing rules, healthcare professions without a full registration system base their practice largely upon society-based professional bodies (Table 3).

**Table 3:** Scope of practice and code of ethics provided by professional genetic counselling organsations

Region	Professional organisation	Official website
United States of America^a,b^	National Society of Genetic Counselors (NSGC)	https://www.nsgc.org/
United Kingdom^a,b^	Genetic Counsellor Registration Board (GCRB)	https://gcrb.org.uk/
Europe^a,b^	European Board of Medical Genetics (EBMG)	https://www.ebmg.eu/
Australia and New Zealand^a,b^	Human Genetics Society of Australasia (HGSA)	https://www.hgsa.org.au/
India^a,b^	Board of Genetic Counseling of India (BGC)	http://www.geneticcounselingboardindia.com/
South Africa^a^	Southern Africa Society of Human Genetics (SASHG)	https://sashg.org/
Canada^b^	Canadian Association of Genetic Counsellors	https://www.cbgc-cccg.ca/

^a^With published scope of practice

^b^With published code of ethics

The US has been the leading pioneer in the development of the GC profession since the establishment of the NSGC in 1979 (ref. [15]), which has played a crucial role in shaping the profession. The United Kingdom (UK) established the Association of Genetic Nurses and Counsellors (AGNC) in the late 1980s, linking accredited local training programmes to their registration system – the Genetic Counsellor Registration Board (GCRB), which transitioned under the auspices of the Academy of Healthcare Sciences (AHCS) in 2019, and is now represented on the Academy’s Healthcare Science Registration Council [16, 17]. In Europe, the European Board of Medical Genetics (EBMG) was established in 2012 and became a legal entity in 2014 (ref. [18]). In Africa, the Southern African Society for Human Genetics was formed in the early 1990s, while the Australasian Society of Genetic Counsellors was founded in 1993. In Asia, the Board of Genetic Counseling of India (BGC) was registered with the government of Telangana in 2015 (ref. [19]). Israel initiated its GC profession with the first master’s training program in 1997 (ref. [20]). Unlike most of the self-regulation systems in Western countries, Israel and Saudi Arabia in the Middle East have their own national GC regulation systems. However, albeit licensed, genetic counsellors in Israel are not recognised as an independent profession, and therefore, must work under the supervision of a medical geneticist [21]. In South Asia, Taiwan formed the Taiwan Association of Genetic Counselling in 1997 (ref. [22]), and Japan founded the Japanese Society of Genetic Counselors in 2001 (ref. [23]). The development of the GC in other regions, such as South Korea, the Philippines, Singapore, Thailand and Malaysia, is still in its infant stages. Mainland China is also in the very preliminary of GC development, but recent strong support from the Central Government has inspired breakthroughs in the field, including the founding of the Chinese Board of Genetic Counseling in 2015 to provide basic short-term training courses. However, without a systematic training framework, services provided by quasi-genetic counsellors in the region vary [24].

### Consistency, diversity and controversies in the scope of practice for genetic counselling

In this global review and analysis, a broad consistency of practice duties is found, with some minor differences in practice due to social-cultural influences in different regions. In general, genetic counsellors around the globe adopted a similar counselling protocol, in which they build rapport and obtain informed consent; collect family and medical histories from the patients (and their families); assess and discuss potential risks for genetic conditions before genetic testing. An in-depth analysis of the information published also revealed some diversities in the approaches of genetic counsellors in interpreting and communicating the genetic test results with the patients; promoting psychological adaptations; and referring patients to further care when needed in post-test counselling sessions. Ordering genetic molecular tests, however, remains a controversial issue in many countries. Such ambiguity in GC practice poses potential implications not only for delineating the role of genetic counsellors but also for clinical application and health economics.

Language such as “identify,” “coordinate” and “facilitate” appropriate genetic and/or genomic testing and other investigations have been used in the SOP provided by the six professional bodies. Other than the 13 states that explicitly allowed/prohibited genetic counsellors from ordering tests [25], this may have allowed GC practitioners in other states, even without licensure law in the US, to order tests in the past, although one could argue the opposite before the SOP provided by NSGC was revised to allow genetic counsellors to “identify, order and coordinate” genetic laboratory tests [15]. Similar to the majority of states in the US, genetic counsellors in the UK and laboratory genetic counsellors working in Australia and New Zealand can order tests independently [13, 16]. Working with clinical geneticists, genetic counsellors in Israel and Africa can also order tests for their clients if needed [21, 26]. However, medical geneticists would usually order genetic tests; and, supervision or a co-signature from a clinical geneticist is often required for ordering tests, according to genetic counsellors practising in Canada [27] and Europe [28]. While the role of genetic counsellors in ordering tests is not specified in most of the Asian regions, the government in Singapore has emphasised that ordering tests should be done by registered medical practitioners, and not genetic counsellors, as their main duty is to provide GC services [29].

Ordering tests to diagnose or manage medical conditions is a process that requires genetic and/or genomic knowledge and coordination, hence, relying on medical geneticists or trained and experienced genetic counsellors to calculate risk, analyse inheritance patterns and select the most cost-effective and clinically appropriate tests [30, 31]. Due to the limitations in the SOP discussed, or following the routine GC practice, only 21.3% of genetic counsellors in Europe ordered genetic tests without other medical geneticists [28], and 26.2% in Australia ordered whole exome sequencing or whole genome sequencing [32]. However, a US study found that genetic tests were often mis-ordered, at an average of 61% per month, resulting from the 26% of requests for genetic test orders changed by genetic counsellors [33]. Such a high frequency of mis-orders could be due to some current practices in clinical settings. For example, a co-signature from a physician is needed to order a genetic test in Canada, although the physician did not meet the patient, and only learnt about the patient’s medical condition based on the letter from the genetic counsellor [27]. While test orders reviewed and facilitated by genetic counsellors could reduce costs to the healthcare system and patients, saving up to $48,000 per month [33], it is paramount to ensure the appropriate ordering of genetic tests, and educate non-geneticist practitioners and other healthcare professionals about genetic and genomic testing and associated concepts, as the integration of genetic testing into healthcare increasingly relies on multidisciplinary teams in this genomic era [34, 35].

### Striving towards a shared decision-making model in genomic medicine

One primary and unique component of GC is the element of counselling, in this context, which may refer to the process of information-giving or communication as opposed to psychotherapy. While GC in the North America, Europe, UK, Australia and South Africa adopted a patient-centred psychotherapeutic process, process studies show that a didactic, teaching model-based process was preferred in the US, Canada and China [36–39]. In the currently dominant non-directive model of GC, the genetic counsellor provides support and guidance through the session on the diagnosis, natural history and inheritance of the genetic condition on a patients-centred based model, as it receives better patient outcomes compared to an information-based teaching approach [40]. In such an informed decision-making model, as much as the help provided by the genetic counsellor, the patient is required to reach his/her decision, rather than relying on the genetic counsellor in decision making. However, this process demands the patient’s competencies, such as knowledge, understanding and experiences, to balance the benefits and risks in order to reach a wise decision [41]. Given that genomic medicine is a relatively new topic, and its education system is yet to mature, genomic illiteracy could pose a challenge for decision-making, or even a high risk for erroneous decisions. The shared decision-making model allows genetic counsellors and clients to share information, reaching the best decision with them after integrating the needs, values and emotions of the client [41, 42], and therefore, has been considered and could be promoted to a global scale [43].

### Ethical considerations in genetic counselling

Other than the SOP, the COE is another important document usually exist and adopt side by side with the SOP. Making reference to the published COEs in the four pioneering countries, i.e., US [44], UK [45], Canada [46] and Australasia [47], the most important ethical considerations for genetic counsellors are discussed. Complementary to the SOP, the COE acknowledges the importance of ethical conduct in four major areas of responsibility: towards themselves, their colleagues, clients and society. Personal responsibilities highlight the qualities and duties genetic counsellors value in themselves, emphasising integrity, competence, professionalism, veracity, accountability, dignity and self-respect. Maintaining, continuing, and improving one’s own professional education and competence is regarded as critical to staying informed on pertinent rules, policies, stances and standards for the practice of GC. Working with other colleagues, genetic counsellors should share their knowledge and experience for the wellbeing and benefit of clients and carers. Based on mutual respect, cooperation, support and caring, the relationship with colleagues should enhance the quality of service [48].

Responsibilities of genetic counsellors towards their clients focus on addressing their needs and expectations based on duties of care and respect for their autonomy, individuality, welfare and freedom. This respect is fundamental in fostering trust and ensuring that clients feel empowered in their healthcare choices [48]. Much emphasis was placed on the protection of patients’ privacy and confidentiality. While NSGC adopts a general approach, stating that genetic counsellors should maintain patients’ privacy and confidentiality unless “the disclosure is required by law,” AGNC is more stringent and affirms that disclosures should only be made with the consent of the service users, unless “it can be justified because of a significant risk to others.”[49] Under the system of common law, as in the US, UK (until the case of *ABC v St George’s Healthcare NHS Trust and others*^1^) and Australia, disclosure of genetic information is permitted but not obligatory if the advantages exceed the drawbacks. The judge in this case concluded that medical professionals, particularly those are with a close proximal relationship with the at-risk person, owe a legal duty to balance the rights and interests of another party and the public against the patient’s consent for prevailing confidentiality, when the disclosure involves a genetic relative whose risk of significant harm could be reduced by it [50]. This final verdict provides compelling evidence in Hong Kong that healthcare professionals have a duty of care that extends to performing the balancing exercise, and acting in accordance with the mutual benefits of their patients and any at-risk third parties [51]. In France, unlike the UK, patients have a unique legal obligated role to inform their family members of a significant and curable hereditary risk; they can either do this directly or delegate the responsibility to medical professionals. Genetic counsellors are encouraged to engage in ongoing professional education to stay informed about evolving practices and legal requirements related to confidentiality and patient rights [48].

For the public, genetic counsellors should work to advance the general welfare of society. This includes promoting public education, preventing genetic discrimination, promoting change in the field of GC, and advocating for equitable access to healthcare. Genetic counsellors should incorporate the latest research and advancements into their practice in their field, and raise awareness of new developments in genetic testing and counselling to the general public. For example, genetic counsellors should be well-prepared to accommodate the increasing demands of service in modern family structures, including sperm donors, surrogate mothers and sexual minority populations [52]. First, they have to be familiar with the updates, such as those in 2008 (ref. [53]) and 2016 (ref. [54]), to represent the non-binary gender identities in the pedigree at the patient-care levels. Subsequently, they could work toward aligning the practice regarding appropriate nomenclature that would be the pedigree’s most precise and concise interpretation to represent their patients’ gender identities [55]. One should aim at enhancing professionalism through consistency and competence, serving as an important source of reliable information and expert opinion on GC for relevant stakeholders. Genetic counsellors, thus, play a crucial role in preventing genetic discrimination by advocating for policies and laws that protect individuals from being discriminated against based on their genetic information [52]. In addition, they encourage legislation that aims to stop genetic discrimination and eliminate the use of genomic information as a claim for any form of discrimination.

## Scope of practice and code of ethics endorsed by Hong Kong Genetic Counselling Practice Consortium

Through the present work of reviewing global GC practice on top of extensive clinical experiences in the field, the authors acknowledged that GC development is a dynamic process, evolving as technologies and demands change with time. Moving on to the genomic era, GC practices do not only focus on clinical settings, but a significant number of genetic counsellors have also begun to work in more diverse settings, such as being team members of different medical specialties, most frequently obstetrics, paediatrics, oncology, cardiology and neurology, in genetic testing laboratories, in research departments, in commercial companies and clinics, and with government agencies. Such changes have been reflected in the development of HGSA’s SOP, which has expanded to cover the roles of six types of genetic counsellors – clinical, industry, academic, policy, research and education - in its recent version delivered in 2022 (ref. [13]). Consistent with Australia and New Zealand, the SOPs in the US and UK have been revised to accommodate the changes resulting from the evolution from genetics to genomics. While the GCRB summarised the role of genetic counsellors into 16 areas in 2021 from a long statement published with ANGC and AHCS in 2020 (ref. [16, 56]), NSGC made a minor update in their SOP recently, allowing genetic counsellors to order genetic laboratory tests [15], in addition to identifying and coordinating them in 2012 (ref. [57]). To capture the current landscape and the dynamic development of genomic medicine in Hong Kong, the Consortium adopted a similar process in formulating the SOP in the local setting after multiple rounds of robust and constructive deliberation.

### BRIDGE – the depth and width of the scope of practice for genetic counsellors in Hong Kong

**Fig. 3:**
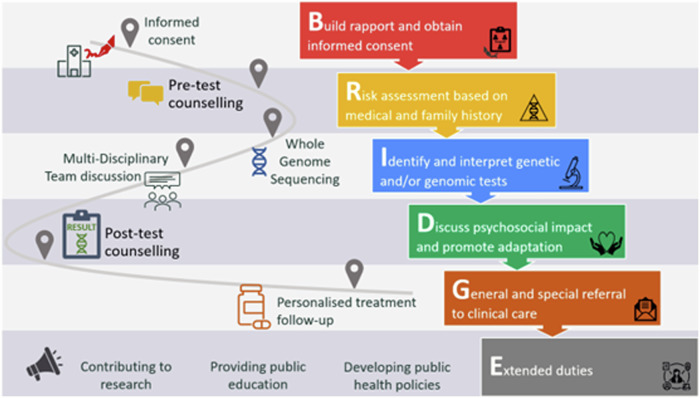
Mapping Hong Kong Genome Project to the scope of practice in local settings, highlighting key phases and actions in the workflow

It is pivotal to align and standardise GC practices covered by a significant number of personnel providing GC services in Hong Kong. The SOP for Hong Kong genetic counsellors is formulated largely upon the results of the conclusions drawn from the thematic analysis of GC practice in the Americas, Europe, Oceania, Africa and Asia, taking into considerations the unique cultural and professional characteristics of the industry in Hong Kong. Mapping also to the patient’s journey of HKGP as a well-established genomic counselling and testing model [11], the authors coined the BRIDGE mnemonic to summarise the key areas of practice duties for genetic counsellors working in clinical settings (Fig. 3). It also aids in strategic marketing, and makes the concepts easier for genetic counsellors and other stakeholders to understand and remember. Using the HKGP as a service model, trained personnel with the role of a genetic counsellor working in the Partnering Centres are heavily responsible for building rapport with the potential participant; introducing the study details; and obtaining informed consent for the research study. During pre-test counselling, they collect patients’ medical and family histories to assist in the risk calculation and assessment process. Upon receiving the biological sample, DNA is extracted for WGS. After bioinformatic analysis of WGS and genomic variant interpretation, a multi-disciplinary team meeting took place, where the genetic counsellor also has a role in interpreting and discussing the ‘phenotypic fit,” WGS findings, and any need for further investigation with other clinical and medical professionals. The genetic counsellor then communicates and further discusses the results, particularly regarding the psychosocial impact on patients and families to understand and adapt to conditions diagnosed by genomic technologies or related to genomic medicine in the post-test counselling sessions. When there is a need, the patients will be referred for further clinical or psychological follow-up. In addition, experienced or advanced genetic counsellors should also take up extended duties such as addressing service gaps through research; educating other clinical and allied health professionals, as well as the general public; and some of them participated in developing healthcare policy with the HHB (i.e., Strategic Development of Genomic Medicine in Hong Kong) [9]

The COE developed and endorsed alongside the SOP is based upon the relationships genetic counsellors have with their core selves, clients, colleagues and the community (the four C’s of conduct). Each major section of the COE begins with a description of one of these relationships, along with some of its values and characteristics. These values are drawn from the overarching principles of medical ethics: autonomy, beneficence, non-maleficence and justice. Genetic counsellors are expected to uphold these principles and act ethically in all professional relationships, as stipulated. This COE is intended to provide a direction for the conduct of anyone who may best serve the aspirations and values of GC. Like other guidelines, the COE cannot and does not serve to provide all the assistance and guidance needed in every clinical situation, especially when various relationships may appear to conflict in real life scenarios. Therefore, when considered appropriate for the COE, guidelines for prioritising the relationships have been stated. For example, genetic counsellors should strive to maintain the privacy and security of the confidential information received from clients and families, considering appropriate legal requirements for disclosure. Some opacity and ambiguity may remain in other areas, allowing for the experience of genetic counsellors to properly respond to challenging situations. When such situations arise, it is crucial for the genetic counsellor concerned to discuss the case with their supervisors and/or other experts in genomic medicine. The Consortium keeps the COE under continuous review. The Code was developed with input from a variety of sources, including international practices, local peer opinion, legal requirements, public expectations and moral commitments.

## Implications for other regions facing similar challenges

The review findings and Consortium’s initiatives in Hong Kong stand out in several key ways when compared to international practices, highlighting the originality of our approach. One notable difference is the emphasis on contextualising GC roles within the local healthcare landscape that integrates cultural, social and healthcare nuances specific to Hong Kong. Unlike many established SOPs from organisations such as the NSGC and HGSA, which primarily address traditional clinical settings, our findings indicate a significant shift in Hong Kong towards recognising the diverse environments in which genetic counsellors operate, including research, industry and public health. Other regions may adopt this approach by fostering partnerships across healthcare disciplines, ensuring that genetic counsellors are integrated into multidisciplinary teams. This not only improves patient care by providing comprehensive support but also enhances the visibility and relevance of GC services within the broader healthcare system.

Our approach of formulating the SOP and COE through the thematic review and collaborative development process also provides a framework that could inform practices in other regions facing similar challenges. By conducting thorough reviews of existing practices, and engaging local stakeholders, areas with different cultural attitudes towards genetics can develop guidelines that resonate with their populations, ultimately enhancing the effectiveness and acceptance of GC services. While international practices have begun to revise their frameworks to accommodate advancements in genomics, our review suggests that Hong Kong’s approach is particularly nimble, reflecting a proactive engagement with the rapid changes in genomic medicine. The collaborative development process allows for a more agile response to the rapidly changing landscape of genomic medicine. This adaptability ensures that the guidelines are relevant to the complexities of modern healthcare, ultimately leading to improved patient outcomes, and a more integrated healthcare approach.

## Conclusion

Learning from the experiences of the overseas and local pioneers of GC development, the Consortium is formed to initiate the important work of reviewing, monitoring, and enhancing GC services that facilitate the process of disease diagnosis and management to benefit patients in the genomic era. Genetic counsellors should also aspire to become a pivotal part of modern precision medicine by helping physicians make good use of the genomic databases established for various diseases, and bridging communication gaps between physicians, patients and members of the public. With the rapid advancement of genomic technologies and their application to medicine, the practice of GC is also expanding and evolving from earlier approaches. Seasoned genetic counsellors who received initial training for over ten years may urgently need to undergo further and updated training to adapt to the new advancement of the industry. In view of the pressing need to match up with the services provided around the globe, the SOP of GC in Hong Kong is mapped out by using thematic analysis as an important initial step so that we may weave it into the context of the development of this profession worldwide while maintaining our uniqueness as a community with a distinct societal culture. Next, the Consortium aims at issuing the standard guidelines, and educating important stakeholders in the industry and potential employers about the SOP. Planning and work in supporting the local genetic counsellors in their professional training to meet the standards of the SOP and COE also need to be executed in phases. Engaging the local population to find out their educational needs, and identify misconceptions in the community is also critical to the GC development. The end products will help increase genetic/genomic awareness and literacy. The profession of genetic counselling is a complex and ever-evolving field. Under the direction laid down by the concerted efforts and consensus of the Consortium, genetic counsellors will have a clear SOP, allowing their performance reviews to be accurately measured against appropriate benchmarks. This solid step forward will help genetic counsellors in Hong Kong be well received in various workplaces, including hospitals, research institutions, genetic testing laboratories, government agencies and private companies, contributing significantly to the health of the people of Hong Kong.

## Supplementary information

Appendix 1 and 2

Supplementary table S1

## Footnotes

1 The claimant (known as ABC to protect her child) is the daughter of a man with Huntington's disease diagnosed when he was seized under the Mental Health Act at a hospital run by a trust for killing his wife. During the detention, family therapy sessions were arranged by the trust for both the father, who was a patient in the hospital, and ABC as a victim of her father’s crime. When the medical team consulted ABC’s father regarding the disclosure of his genetic disorder, which has a 50% risk of passing down to the next generation, he objected. Thus, his diagnosis was not disclosed to ABC, who was pregnant. Subsequently, after testing, ABC unintentionally learned that she shared her father's aberrant Huntington's gene. ABC filed a lawsuit for negligence against the medical staff for not informing her of the genetic risk. She claimed that the medical team owed her a "duty of care" (a legal obligation to take her rights and interests into account) that should have prevailed over her father's interest and the public interest in maintaining his confidentiality in this situation. After the initial ruling was overturned by the court, the judge concluded that the medical professionals owe a legal duty to consider the rights and interests of another party and the public other than the patient’s consent in prevailing confidentiality, when the disclosure involves a genetic relative whose risk of significant harm could be reduced by it.
