# Supplementary material for: A step forward in genetic counselling: defining practice and ethics through the Genetic Counselling Practice Consortium in Hong Kong
Source: J Hum Genet. 2025 Mar 14;70(5):233–41. doi: 10.1038/s10038-025-01321-5 (PMC11964909; doi:10.1038/s10038-025-01321-5)
Supplement: Supplementary file 1 — Appendix 1 and 2 [file 10038_2025_1321_MOESM1_ESM.docx]

**Appendix 1**

**Scope of Practice for Genetic Counsellors**

This **Scope of Practice for Genetic Counsellors** is relevant to their roles undertaking direct patient care, as well as roles in research, education, management, policy, and strategy. When serving their patients, genetic counsellors should uphold high standards of the scope of practice. The key areas of genetic counselling practice are summarised in the mnemonic: “BRIDGE” as follows:

**B**uild rapport and obtain informed consent:

- Utilise counselling and communication skills to build good rapport with patients for better communication and understanding
- Obtain informed consent for appropriate genetic/ genomic tests and other diagnostic studies as relevant for the genetic assessment; and
- Deliver and discuss information relevant to the benefits, risks, limitations, and confidentiality of genetic testing.

**R**isk assessment based on medical and family history:

- Gather and interpret patient and family information, including medical history, family history, and prior genetic work-up, to construct and update the pedigree in a relevant, succinct, and logical manner;
- Identify how genetics contributes to the patients’ condition, and assess the risk of inheritance in specified relatives or individuals; and
- Comprehend and assess the characteristics, natural history, means of diagnosis, genetic and environmental factors, and management of risk for genetic or medical conditions.

**I**dentify and interpret genetic and/ or genomic tests:

- Integrate and interpret the information collected with the latest medical information about genetic disorders to identify, coordinate and facilitate the most appropriate genetic/ genomic test for the patient and family;
- Understand the implications of genetic tests and technologies for management options for the disorder and its recurrence; and
- Interpret test findings with other members of the Multidisciplinary Team to provide optimal services for patients.

**D**iscuss psychosocial impact and promote adaptation:

- Make psychosocial assessment of patient need, provide patient support to navigate and adapt to the implication of genetic health information;
- Create a safe and comfortable environment to support the expression of the patient’s emotions/ feelings, concerns, expectations, motivations, beliefs and ethical issues raised; and
- Use therapeutic counselling frameworks, skills, and communication strategies to facilitate informed decision-making for patients and families, enabling informed choices, and understanding of the implications of those choices.

**G**eneral and special referral to clinical care:

- Ensure that patients have access to the full range of resources, services and decision options;
- Maintain clear, concise, and accurate documentation for families and other healthcare professionals; and
- Make timely, appropriate referrals to other healthcare professionals and community resources as appropriate.

**E**xtended duties

- Contribute to research and translate findings into evidence-based practice;
- Proactively engage in regular counselling supervision that encourages self-reflection and the continued development of professional practice;
- Advocate for the profession of genetic counselling by modelling high standards of personal and professional behaviour in all interactions;
- Serve as a genetic healthcare resource for professionals and patients (and their family members);
- Plan, organise and deliver education in genetic healthcare for professionals and the public; and
- Participate in the evaluation and development of guidelines, regulations and policies that may influence wellbeing, clinical practices and health services, aiming to improve standards of service delivery of genetic/ genomics in healthcare settings.

**Appendix 2**

**Code of Ethics for Genetic Counsellors**

This **Code of Ethics for Genetic Counsellors** is established to affirm the ethical responsibilities of genetic counsellors (or any professionals serving the same function) and provide guidance in their relationships with core self, clients, colleagues, and community (four C’s of conduct).

Genetic counsellors (and their equivalents) in Hong Kong are expected to be aware of the ethical and moral implications of their professional actions, and to comply with the guidelines and principles set forth in this code.

**1. Responsibility towards the Core self**

Genetic counsellors value the importance of clinical competence, integrity, veracity, dignity, and self-respect in their core selves as well as in each other. Genetic counsellors also have the responsibility to practise continuous self-evaluation and reflection to maintain a high standard of practice. Therefore, genetic counsellors should strive to:

- Pursue and acquire sufficient, relevant, accurate, and up-to-date information required for a given situation.
- Engage in continuous education, specific training and development as necessary to provide the highest standard of professional practice.
- Keep abreast of current standards of practice, relevant guidelines, and regulations.
- Recognise the limits of their own knowledge, expertise, and therefore competence and limits in any given situation; and refer their patients to other reliable and qualified professionals when they are not able to provide necessary and suitable support.
- Correctly represent their experience, competence, and credentials, including training, academic degrees, and relevant work experiences.
- Acknowledge and disclose circumstances that may result in any possible conflict of interest.
- Avoid relationships and activities that interfere with professional judgement or objectivity.
- Be responsible for their own physical and emotional health and wellbeing, and the impact on their professional performance.

**2. Responsibility towards their Clients**

The Genetic Counsellor-Patient relationship is based on values of care and respect for the client’s autonomy, individuality, welfare, and freedom. The major concern of genetic counsellors is the interests of their patients. According to Veach et al. (2003), acting as a professional means responsibly “addressing patients’ needs and expectations.” This requires first “an understanding of yourself and the personal characteristics that you bring with you into genetic counselling sessions” that may affect the counselling experience of the clients. Therefore, genetic counsellors should strive to:

- Serve eligible clients who seek services regardless of personal or external interests or biases.
- Enable their clients to make informed decisions free of coercion, by providing or illuminating the necessary facts, clarifying the alternatives and describing the anticipated consequences in details throughout the entire decision-making process.
- Consider the clients’ best interests in any decision making.
- Respect their clients’ beliefs, preferences, circumstances, feelings, family relationships, and cultural traditions.
- Refer clients to other qualified professionals if they are certain that they lack specific capacities to assist the clients.
- Maintain strict confidentiality regarding medical and genetic information obtained from clients and families. Exceptional disclosure is considered only when non-disclosure may cause risk of serious harm to others.
- Clarify and define their professional roles and relationships with clients, and provide an accurate description of their services.
- Be aware and sensitive about how the clients’ culture, motives, and values may be brought into the counselling sessions.
- Avoid the exploitation of their clients for personal advantage, profit, or interest.

**3. Responsibility towards their Colleagues**

Genetic counsellors often provide services by closely working with other healthcare professionals. The relationships among genetic counsellors or with other colleagues are based on mutual respect, caring, cooperation, and support. Genetic counsellors should strive to:

- Respect and value the knowledge, perspectives, contributions, and areas of competence of their colleagues, and collaborate with the team in providing the highest quality of service.
- Share their knowledge and provide necessary support for the development of other genetic counsellors and the genetic counselling profession.
- Assure that individuals under their supervision undertake responsibilities that are equivalent to their knowledge, experience, and training.
- Encourage ethical behaviours among colleagues.
- Take responsibility or credits only for work that they have performed or contributed; and acknowledge the work and contributions of others.
- Maintain appropriate boundaries to avoid exploitation in their relationships with colleagues.

**4. Responsibility towards the Community**

The relationships of genetic counsellors with our community and society include interests and active participation in activities that promote the well-being of the people, including access to genetic/genomic services. Therefore, genetic counsellors should strive to:

- Promote the equality of individuals and advocate for equity of access to healthcare, especially in genetic services.
- Promote policies that aim to prevent genetic discrimination and oppose the use of genetic information as the basis for discrimination of any sort.
- Participate in activities necessary to promote socially responsible change in the field of genetic counselling services.
- Serve as an important source of reliable information and expert opinion on genetic counselling for relevant stakeholders.
- Promote public awareness of potential social impact of advances in genomic science and technology.
