# Supplementary material for: A step forward in genetic counselling: defining practice and ethics through the Genetic Counselling Practice Consortium in Hong Kong
Source: J Hum Genet. 2025 Mar 14;70(5):233–41. doi: 10.1038/s10038-025-01321-5 (PMC11964909; doi:10.1038/s10038-025-01321-5)
Supplement: Supplementary file 2 — Supplementary table S1 [file 10038_2025_1321_MOESM2_ESM.docx]

**Supplementary table S1. Member list of the Hong Kong Genetic Counselling Practice Consortium**

|  | **Name** | **Affiliation** |
| --- | --- | --- |
| **Co-chairs** | **Mr Sam Hui** | Deputy Secretary for Health 1, Health Bureau |
|  | **Dr Su-vui LO** | Chief Executive Officer, Hong Kong Genome Institute |
| **Executive Members** | **Dr Derrick Kit Sing AU** | Honorary Advisor, CUHK Centre for Bioethics, The Chinese University of Hong Kong |
|  | **Prof Dennis Yuk Ming LO** | Director of the Li Ka Shing Institute of Health Sciences, the Li Ka Shing Professor of Medicine and Professor of Chemical Pathology of The Chinese University of Hong Kong |
|  | **Dr Calvin Wai Loon HO** | Co-Director, Teaching Faculty & Research Fellow, Centre for Medical Ethics and Law, The University of Hong Kong |
|  | **Prof Ava Kwong** | Clinical Professor, Department of Surgery, School of Clinical Medicine, The University of Hong Kong |
|  | **Dr Wendy Wing Tak LAM** | Division Head, Division of Behavioural Sciences, The University of Hong Kong |
|  | **Dr Mary Hoi Yin TANG** | Clinical Associate Professor, The University of Hong Kong |
|  | **Dr Ivan Fai Man LO** | Head of the Clinical Genetic Service, Department of Health |
|  | **Ms Priscilla Yee Hung POON** | Chief Manager (Allied Health), Cluster Services Division, Hospital Authority |
|  | **Ms Vivian Shuk Man CHAN** | Manager (Nursing), Nursing Services Department, Hospital Authority |
